# THAOS: Gastrointestinal manifestations of transthyretin amyloidosis - common complications of a rare disease

**DOI:** 10.1186/1750-1172-9-61

**Published:** 2014-04-27

**Authors:** Jonas Wixner, Rajiv Mundayat, Onur N Karayal, Intissar Anan, Pontus Karling, Ole B Suhr

**Affiliations:** 1 Department of Public Health and Clinical Medicine, Umeå University, Umeå S-901 87, Sweden; 2 Pfizer Inc, New York, NY, USA

**Keywords:** Amyloid, Amyloid neuropathies, Amyloidosis, Hereditary, Cardiomyopathies, Secondary, Gastrointestinal disorders, Functional, Nutritional status, Quality of life, Transthyretin

## Abstract

**Background:**

Transthyretin amyloidosis is a systemic disorder caused by amyloid deposits formed by misfolded transthyretin monomers. Two main forms exist: hereditary and wild-type transthyretin amyloidosis, the former associated with transthyretin gene mutations. There are several disease manifestations; however, gastrointestinal complications are common in the hereditary form. The aim of this study was to explore the prevalence and distribution of gastrointestinal manifestations in transthyretin amyloidosis and to evaluate their impact on the patients’ nutritional status and health-related quality of life (HRQoL).

**Methods:**

The Transthyretin Amyloidosis Outcomes Survey (THAOS) is the first global, multicenter, longitudinal, observational survey that collects data on patients with transthyretin amyloidosis and the registry is sponsored by Pfizer Inc. This study presents baseline data from patients enrolled in THAOS as of June 2013. The modified body mass index (mBMI), in which BMI is multiplied with serum albumin, was used to assess the nutritional status and the EQ-5D Index was used to assess HRQoL.

**Results:**

Data from 1579 patients with hereditary transthyretin amyloidosis and 160 patients with wild-type transthyretin amyloidosis were analyzed. Sixty-three percent of those with the hereditary form and 15% of those with the wild-type form reported gastrointestinal symptoms at enrollment. Unintentional weight loss and early satiety were the most frequent symptoms, reported by 32% and 26% of those with transthyretin gene mutations, respectively. Early-onset patients (<50 years) reported gastrointestinal complaints more frequently than those with a late onset (p < 0.001) and gastrointestinal symptoms were more common in patients with the V30M mutation than in those with other mutations (p < 0.001). For patients with predominantly cardiac complications, the prevalence of gastrointestinal manifestations was not evidently higher than that expected in the general population. Both upper and lower gastrointestinal symptoms were significant negative predictors of mBMI and the EQ-5D Index Score (p < 0.001 for all).

**Conclusions:**

Gastrointestinal symptoms were common in patients with hereditary transthyretin amyloidosis and had a significant negative impact on their nutritional status and HRQoL. However, patients with wild-type transthyretin amyloidosis or transthyretin mutations associated with predominantly cardiac complications did not show an increased prevalence of gastrointestinal disturbances.

## Background

Transthyretin amyloid (ATTR) amyloidosis is a systemic disorder with two main forms; hereditary and wild-type (wt) ATTR amyloidosis. The hereditary form is caused by transthyretin (TTR) gene mutations and is sometimes referred to as familial amyloid polyneuropathy (FAP) or familial amyloid cardiomyopathy (FAC). Wt-ATTR amyloidosis is also known as senile systemic amyloidosis (SSA) or senile cardiac amyloidosis (SCA), due to its late onset and primarily cardiac manifestations.

Hereditary ATTR amyloidosis is a rare autosomal dominant condition that is present all over the world with endemic areas in Sweden, Portugal, Brazil and Japan. More than 100 different TTR mutations have been observed, however, the V30M (Valine substituted for Methionine at position 30) mutation is the most common form in endemic areas [1,2]. The V122I mutation is probably the most common variant worldwide with a prevalence of about 4% in African-Americans [3].

TTR is a tetrameric protein that functions as a transporter of thyroxin and retinol binding protein and is mainly produced by the liver [4,5]. Increasing age and amyloidogenic TTR mutations lead to a decreased stability of the TTR tetramer, making it more prone to dissociate into non-native monomers. These monomers, in turn, aggregate into beta structured fibrils that form extracellular amyloid deposits [6,7].

ATTR amyloidosis is often classified as polyneuropathic, cardiac or mixed type, depending on the clinical appearance. In addition, oculoleptomeningeal and leptomeningeal types with symptoms of eye and central nervous system malfunction have been described [8-12]. However, this is an entirely artificial classification since more than 50% of all mutations are reported to produce cardiac involvement [13] and for patients with the V30M mutation, cardiac involvement is age dependent with cardiomyopathy predominantly occurring in late-onset cases (≥50 years of age).

In hereditary ATTR amyloidosis, GI disturbances play an important role in the patients’ morbidity and mortality [14,15]. Virtually all Swedish V30M patients develop GI complications during the course of the disease [16], whereas GI symptoms are less frequent for other genotypes [17]. In some cases, GI symptoms are present even before the onset of the peripheral polyneuropathy and initial symptoms are often constipation or nausea and vomiting. Diarrhea can also be the presenting symptom [18,19], and in later stages of the disease the diarrhea usually becomes continuous, often coupled with fecal incontinence and severe malnutrition [16,20].

A liver transplantation ceases the synthesis of mutated TTR and thereby halts disease progression. Liver transplantation is currently the standard treatment for hereditary ATTR amyloidosis; however, not all patients are suitable candidates [15,21-23]. Fortunately, alternative treatments are emerging: tafamidis has been shown to stabilize mutated TTR and to reduce disease progression [24] and has been approved in Europe and Japan. Moreover, the NSAID diflunisal has demonstrated efficacy in a controlled trial [25] and gene therapy, aiming to reduce TTR production, is currently under evaluation in clinical trials [26].

The Transthyretin Amyloidosis Outcomes Survey (THAOS) is a large global, longitudinal, observational patient registry designed to better understand and follow the progression of ATTR amyloidosis. The primary aim of the present study was to explore the prevalence of GI manifestations in SSA and in different types of hereditary ATTR amyloidosis, based on enrollment data from THAOS. A secondary aim was to evaluate the impact of the GI symptoms on the patients’ nutritional status and health-related quality of life (HRQoL).

## Methods

### THAOS

The design and methodology of THAOS has previously been described in detail [17,27].

Briefly, THAOS is an international, longitudinal, observational registry that collects data on the progression of ATTR amyloidosis [28] and is sponsored by Pfizer Inc. The registry was established in December 2007 and is open to adults (≥18 years of age) with ATTR amyloidosis and to asymptomatic TTR-variant carriers. Prior to the enrollment of patients in THAOS, the participating sites obtained approval from their local ethical review board/institutional review board. All patients provided written informed consent and research was in accordance with the Declaration of Helsinki. Eligible patients were consecutively enrolled into THAOS by each of the participating sites. De-identified data obtained during routine clinical practice were entered into THAOS using an internet-based application.

### Clinical symptoms and assessments

Enrollment data collected in THAOS as of 6 June 2013 were used for all analyses. Symptoms were recorded as present or absent at the time of enrollment into THAOS. The duration of symptoms was calculated retrospectively from information provided by the patients at the time of enrollment.

The GI symptoms checklist included the following items: early satiety, nausea, vomiting, constipation, alternating diarrhea/constipation, diarrhea, fecal incontinence and unintentional weight loss. Early satiety, nausea and vomiting were regarded as upper GI symptoms, whereas constipation, alternating diarrhea/constipation, diarrhea and fecal incontinence were regarded as lower GI symptoms.

The modified body mass index (mBMI), in which the BMI (kg/m^2^) was multiplied with serum albumin (g/L) to compensate for edema, was used to assess the patients’ nutritional status. mBMI values below 750 kg/m^2^∙g/L were regarded as underweight and values below 600 kg/m^2^∙g/L were regarded as severely malnourished [14,15].

HRQoL was measured using the validated EuroQoL Five Dimensions (EQ-5D) Questionnaire which includes a descriptive and visual analogue scale (VAS) assessment of the present health status. The EQ-5D Index Score was calculated using a predefined scoring algorithm and full health is represented by a score of 1 [29,30].

### Statistical analysis

SAS 9.1.3 (Cary, NC, USA) was used for all analyses. Comparisons between cohorts and subgroups were carried out using the one-way ANOVA for the continuous variables. The chi-square test was used for categorical variables. Multiple regression analyses were carried out to identify potential predictors of GI symptoms, mBMI and the EQ-5D Index Score.

## Results

**Figure 1:**
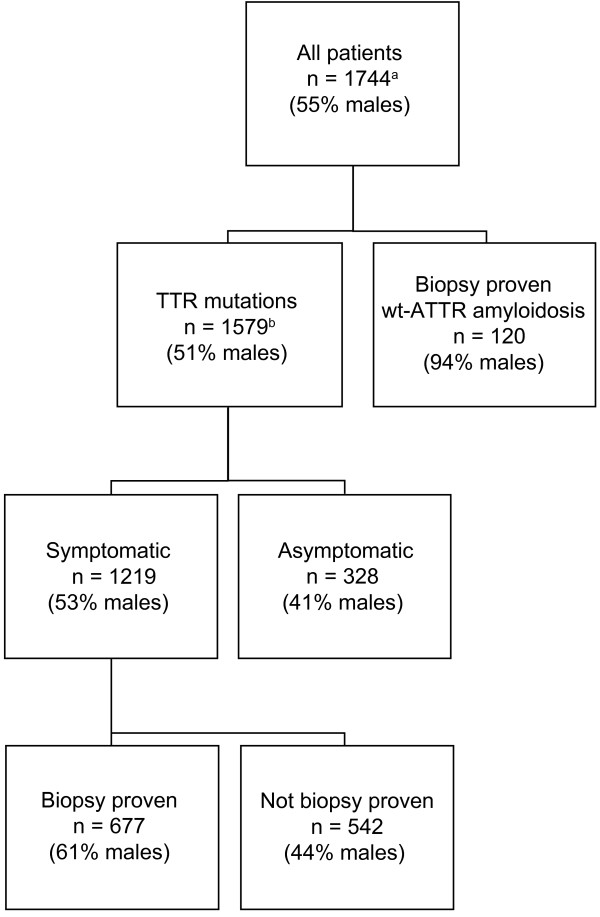
**Characteristics of the patients included in THAOS as of June 2013. **^a^Five patients had gene polymorphisms and 40 patients had a non-biopsy proven diagnosis of wt-ATTR amyloidosis. ^b^Thirty-two patients had an unknown symptomatic status. *ATTR*: transthyretin amyloid; *n*: number of subjects; *THAOS*: transthyretin amyloidosis outcomes survey; *TTR*: transthyretin; *wt*: wild-type.

A total of 1744 patients had been enrolled in THAOS as of 6 June 2013. Seventeen countries participated and the largest contributors were: Portugal (46% of the patients), United States (13%), Italy (7%), France, Germany and Brazil (6% each), Sweden (5%) and Japan (4%). A majority of the patients carried TTR mutations (90.8%), mainly the V30M mutation (73.8%); however, a substantial number of patients had other genotypes or SSA. The most frequent TTR mutations and their associated clinical manifestations are presented in Table 1. Median duration of disease at enrollment in THAOS was 4.9 years for those with TTR mutations. Of the symptomatic patients with TTR mutations, 201 (18%) had undergone liver transplantation prior to enrollment. Further characteristics of the patients included in the analyses are outlined in Figure 1.

**Table 1:** Most abundant TTR mutations and their clinical manifestations

**Mutation**	**Sensory neuropathy**	**Motor neuropathy**	**Gastrointestinal symptoms**	**Cardiac complications**
V30M	707 (89.5%)	305 (38.6%)	547 (69.3%)	212 (26.9%)
V122I	35 (60.3%)	11 (19.0%)	16 (27.1%)	57 (96.6%)
S50R	26 (89.7%)	16 (55.2%)	19 (65.5%)	13 (44.8%)
E89Q	21 (95.5%)	10 (45.5%)	13 (68.4%)	13 (65.0%)
T60A	16 (80.0%)	5 (25.0%)	8 (40.0%)	19 (90.5%)
F64L	18 (90.0%)	11 (55.0%)	10 (50.0%)	7 (35.0%)
S77Y	16 (94.1%)	8 (47.1%)	12 (70.6%)	9 (52.9%)
I68L	7 (46.7%)	6 (40.0%)	2 (13.3%)	13 (86.7%)
I107V	10 (83.3%)	9 (75.0%)	7 (58.3%)	8 (66.7%)
G47A	8 (72.7%)	2 (18.2%)	2 (18.2%)	1 (9.1%)
L111M	1 (10.0%)	0 (0.0%)	1 (10.0%)	7 (70.0%)

Mutations carried by ten individuals or more listed in a descending order.

### GI symptoms

Fifty-nine percent of all the patients in THAOS reported at least one GI symptom. Unintentional weight loss was the most frequently reported symptom (28.3%), followed by early satiety (25.1%) and alternating diarrhea/constipation (22.9%). Fecal incontinence (5.6%) was the least common symptom. The distribution of the individual GI symptoms in patients with hereditary and wt-ATTR amyloidosis is presented in Table 2.

**Table 2:** Distribution of GI symptoms in patients with ATTR amyloidosis

**Symptom**	**Wild-type**	**TTR mutation**
	**n = 140**	**n = 1114**
Any GI symptom	21 (15.3%)	696 (63.0%)
Early satiety	5 (3.6%)	291 (26.4%)
Nausea	3 (2.2%)	189 (17.1%)
Vomiting	0 (0.0%)	147 (13.4%)
Constipation	5 (3.6%)	230 (20.9%)
Diarrhea/constipation	2 (1.5%)	267 (24.3%)
Diarrhea	5 (3.6%)	218 (19.8%)
Fecal incontinence	0 (0.0%)	68 (6.2%)
Unintentional weight loss	4 (2.9%)	346 (31.5%)

Number of patients, n (%), reporting gastrointestinal (GI) symptoms at enrollment.

With regard to genotypic differences, GI complications were found to be less common for some mutations, mainly those associated with predominantly cardiac manifestations (Table 1). Furthermore, GI symptoms were generally more prevalent in V30M than in non-V30M patients (69.3% vs. 56.0%, p < 0.001); (Table 3). Since patients with the TTR V122I, I68L and L111M mutations exhibited mostly cardiac complications and a prevalence of GI symptoms similar to that of the general population; they were excluded from the non-V30M group. For the same reason, subsequent analyses focused on patients with TTR mutations.

**Table 3:** GI symptoms in relation to genotype in patients with TTR mutations

**Symptom**	**V30M**	**Non-V30M**	**p value**
	**n = 792**	**n = 252**	
Any GI symptom	547 (69.3%)	130 (56.0%)	<0.001
Early satiety	242 (30.7%)	44 (19.0%)	<0.001
Nausea	150 (19.0%)	35 (15.1%)	0.172
Vomiting	125 (15.8%)	22 (9.5%)	0.016
Constipation	178 (22.6%)	41 (17.7%)	0.117
Diarrhea/constipation	225 (28.6%)	42 (18.1%)	0.001
Diarrhea	174 (22.1%)	42 (18.2%)	0.199
Fecal incontinence	57 (7.2%)	11 (4.8%)	0.186
Unintentional weight loss	272 (34.6%)	70 (30.3%)	0.224

Number of patients, n (%), reporting gastrointestinal (GI) symptoms at enrollment. The TTR V122I, I68L and L111M mutations (mainly associated with cardiac complications) were excluded from the non-V30M group.

No significant gender related difference in overall GI symptom prevalence was found (females 63.1%, males 62.9%, p = 0.967). However, unintentional weight loss was significantly more common in males than in females (34.9% vs. 27.3%, p = 0.007). Numerical differences in symptom prevalence between men and women were also found for early satiety (28.6% vs. 23.7%, p = 0.067) and nausea (15.2% vs. 19.5%, p = 0.061), but these did not reach statistical significance.

Patients with an early disease onset (<50 years) reported GI symptoms more frequently than those with a late onset (70.3% vs. 49.6%, p < 0.001), yet, no significant differences were observed for constipation and fecal incontinence (Table 4).

**Table 4:** GI symptoms in relation to age at onset in patients with TTR mutations

**Symptom**	**<50 years**	**≥50 years**	**p value**
	**n = 725**	**n = 381**	
Any GI symptom	509 (70.3%)	185 (49.6%)	<0.001
Early satiety	243 (33.7%)	48 (12.9%)	<0.001
Nausea	157 (21.7%)	32 (8.6%)	<0.001
Vomiting	130 (18.0%)	17 (4.6%)	<0.001
Constipation	154 (21.3%)	75 (20.2%)	0.685
Diarrhea/constipation	201 (27.8%)	66 (17.8%)	<0.001
Diarrhea	164 (22.7%)	53 (14.4%)	0.001
Fecal incontinence	51 (7.0%)	17 (4.6%)	0.112
Unintentional weight loss	270 (37.4%)	76 (20.6%)	<0.001

Number of patients, n (%), reporting gastrointestinal (GI) symptoms at enrollment.

**Figure 2:**
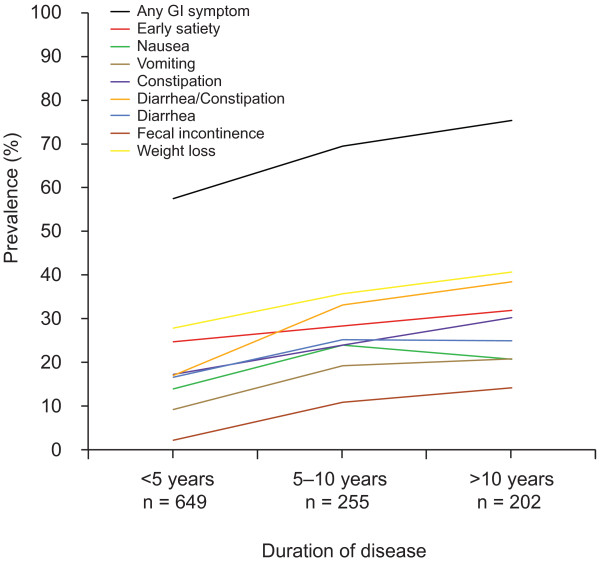
**GI symptoms in relation to duration of disease in patients with TTR mutations.** Prevalence of gastrointestinal (GI) symptoms in patients with a disease duration of <5 years, 5–10 years and >10 years, respectively. A majority of the patients suffered from GI symptoms even at early stages of the disease, whereas the reported prevalence of GI symptoms in the general population usually ranges from 10 to 25%. Unintentional weight loss, early satiety and alternating diarrhea/constipation were the most common symptoms in all disease stages. The symptom prevalence increased significantly with disease duration for all symptoms (p < 0.002, for all), except for early satiety (p = 0.114).

After dividing the patients into groups with a disease duration of <5 years, 5–10 years and >10 years, respectively, the prevalence of GI symptoms was found to be significantly higher in later stages of the disease (57.2% vs. 69.2% vs. 75.1%, p < 0.001). Detailed data on the individual symptoms in relation to duration of disease are presented in Figure 2.

Patients carrying the TTR V30M mutation were included in a multiple regression analysis of factors associated with GI symptoms. Significant associations were found for duration of disease (OR 1.073, 95% CI 1.034-1.114), liver transplantation (OR 4.358, 95% CI 2.460-7.720) and Swedish origin (OR 0.315, 95% CI 0.173-0.572), whereas no significant association was found for male gender (OR 1.322, 95% CI 0.962-1.818), early-onset (OR 1.446, 95% CI 0.996-2.099) or Portuguese origin (OR 1.414, 95% CI 0.973-2.055).

### Nutritional status

**Figure 3:**
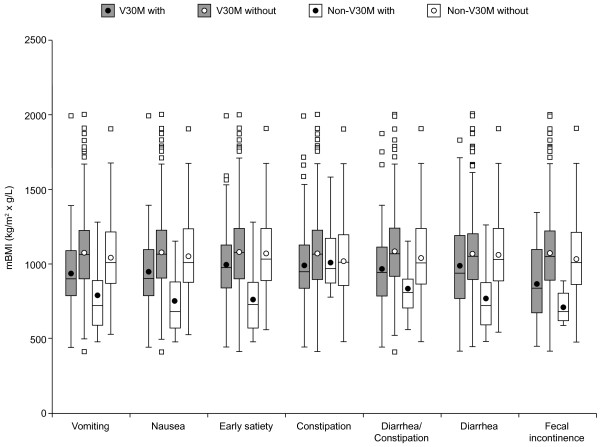
**Impact of gastrointestinal symptoms on the nutritional status in patients with TTR mutations.** For each gastrointestinal symptom, patients with a given symptom were compared with those without the symptom. In the V30M group, each symptom had a significant negative impact on nutritional status, whereas in the non-V30M group, all symptoms but constipation had a significant negative impact. The modified body mass index (mBMI), calculated by multiplying BMI by serum albumin, was used to assess the nutritional status. Extreme mBMI values (<400 or >2500) were excluded and the TTR V122I, I68L and L111M mutations were excluded from the non-V30M group; p < 0.05 was regarded as statistically significant. Data are presented as box and whisker plots showing median (horizontal line), interquartile range (box), mean (circle) and range of measurements (whisker).

The impact of the individual GI symptoms on nutritional status in V30M and non-V30M patients, respectively, is presented in Figure 3. In both groups, patients with a given symptom generally had lower mBMI values compared to those without the symptom.

In addition, the upper and lower GI symptom categories, age at onset, duration of disease and liver transplantation were all significant predictors of mBMI in simple regression analyses. However, in a multiple regression analysis, only the upper and lower GI symptom categories and duration of disease remained significant predictors (Table 5). The outcome was equivalent when the individual GI symptoms were used as independent variables instead of the symptom categories.

**Table 5:** Multiple regression analysis on factors associated with the nutritional status in patients with TTR mutations

**Predictor variable**	**Parameter estimate**	**Standard error**	**t value**	**p value**
Intercept	1214.40	43.12	28.16	<0.001
Gender (male vs. female)	-11.13	17.39	-0.64	0.522
Age at onset (early vs. late)	-34.48	18.96	-1.82	0.069
Disease duration (years)	-3.80	1.34	-2.83	0.005
Upper GI symptoms	-81.42	19.96	-4.08	<0.001
Lower GI symptoms	-98.40	18.91	-5.20	<0.001
LTx at enrollment	-48.53	25.98	-1.87	0.062

The modified body mass index (mBMI), in which BMI was multiplied with serum albumin, was used to assess the nutritional status. Age of onset <50 years was regarded as early-onset. *GI*: gastrointestinal; *LTx*: liver transplant.

### HRQoL

**Figure 4:**
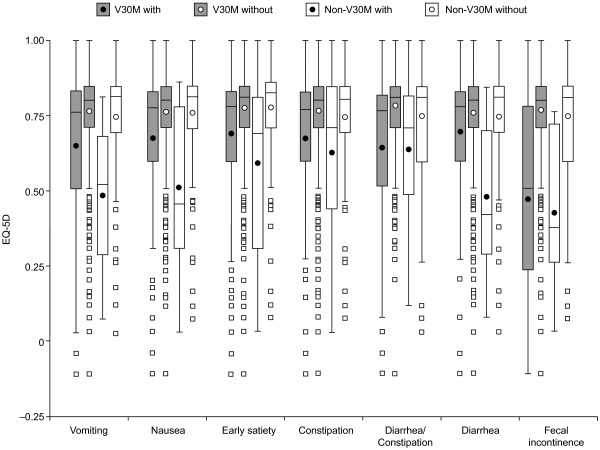
**Impact of gastrointestinal symptoms on HRQoL in patients with TTR mutations.** For each gastrointestinal symptom, patients with a given symptom were compared with those without the symptom. In the V30M group, each symptom had a significant negative impact on health-related quality of life (HRQoL), whereas in the non-V30M group, all symptoms except for alternating diarrhea/constipation showed a significant negative impact. The EQ-5D Index Score was used to assess the HRQoL and full health is represented by a score of 1. The TTR V122I, I68L and L111M mutations were excluded from the non-V30M group; p < 0.05 was regarded as statistically significant. Data are presented as box and whisker plots showing median (horizontal line), interquartile range (box), mean (circle) and range of measurements (whisker).

The impact of the individual GI symptoms on HRQoL in V30M and non-V30M patients, respectively, is presented in Figure 4. As for the nutritional status, patients with a given symptom generally showed reduced EQ-5D Index Scores.

Multiple regression analysis revealed that the upper and lower GI symptom categories, age at onset and duration of disease were all negatively associated with the EQ-5D Index Score, whereas a significant positive association was found for liver transplant at enrollment (Table 6). The outcome remained unchanged when the individual GI symptoms were used as independent variables.

**Table 6:** Multiple regression analysis on factors associated with HRQoL in patients with TTR mutations

**Predictor variable**	**Parameter estimate**	**Standard error**	**t value**	**p value**
Intercept	0.99	0.03	30.53	<0.001
Gender (male vs. female)	0.00	0.01	-0.11	0.909
Age at onset (early vs. late)	-0.09	0.01	-6.19	<0.001
Disease duration (years)	-0.01	0.00	-6.90	<0.001
Upper GI symptoms	-0.07	0.01	-4.77	<0.001
Lower GI symptoms	-0.13	0.01	-9.28	<0.001
LTx at enrollment	0.06	0.02	3.19	0.002

Health-related quality of life (HRQoL) was assessed with the EQ-5D Index Score. Age of onset <50 years was regarded as early-onset. *GI*: gastrointestinal; *LTx*: liver transplant.

Moreover, a weak but significant correlation was found between mBMI and the EQ-5D Index Score (r^2^ = 0.217, p < 0.001).

## Discussion

This is the first detailed analysis of GI manifestations in patients with ATTR amyloidosis, based on data from THAOS, and a large number (n = 1744) of subjects were available for the study.

GI symptoms were generally common and often occurred early after disease onset. However, patients with SSA and TTR mutations associated with predominantly cardiac complications, i.e. the V122I, I68L and L111M variants, reported GI symptoms with prevalence similar to that of the general population (i.e., between 10 and 25% [31-35]). Therefore, subsequent analyses were focused on patients with mainly non-cardiac manifestations.

Unintentional weight loss was the most frequently reported symptom. However, weight loss is multifactorial and a marked loss of weight has been observed in patients with hereditary ATTR amyloidosis even before the onset of GI or other symptoms [36]. The mechanisms behind this weight loss remain unexplained, yet early satiety could be a contributing factor, as it often occurs early in the disease and may negatively affect the patients’ energy intake. An increased metabolism, due to inflammatory reactions and oxidative stress caused by the amyloid formation and deposition [37,38], could also contribute to the loss of weight.

Early satiety was the second most common symptom and early satiety, as well as nausea and vomiting, are classic symptoms of gastric retention, which has been shown to be common in patients with hereditary ATTR amyloidosis [39,40]. As expected, fecal incontinence was the least frequently reported symptom as it occurs in late stages of the disease and as the median duration of disease at inclusion in THAOS was merely 4.9 years.

In the general population, functional GI disorders are more common in females [31-33] and hence, a female preponderance of GI symptoms was expected. However, no significant gender related difference was observed for any GI symptom and there was no association between gender and GI symptoms, nutritional status or HRQoL.

GI symptoms were found to be more frequent in V30M than in non-V30M patients (mutations with mainly cardiac manifestations excluded), which is consistent with previous reports of less frequent autonomic neuropathy in patients with non-V30M mutations [1].

In addition, early-onset patients generally reported GI symptoms more frequently than late-onset cases, which is consistent with previous findings [41,42]. Yet, there was no significant difference in constipation between the groups and this might reflect an early onset of constipation in the late-onset group [19]. The mechanisms behind the phenotypic differences between early and late-onset cases remain unclear, but late-onset cases generally have more cardiac complications and an amyloid fibril composition similar to that of SSA [5].

Since the Swedish and Portuguese V30M populations exhibit phenotypic differences, especially with respect to age at onset and penetrance [43-45], Swedish and Portuguese origin, respectively, were included as predictor variables in a multiple regression analysis of factors associated with GI symptoms. Swedish patients were less likely to suffer from GI symptoms than non-Swedish patients, but no significant association was found between age at onset and GI symptoms, indicating that age is not the factor behind the difference in GI symptom prevalence between Swedish and Portuguese patients.

As expected, the prevalence of GI symptoms was significantly higher for patients enrolled in later stages of their disease. However, it should be noted that as much as 57% of the patients with the shortest disease duration (<5 years) reported GI symptoms. Early satiety was, after unintentional weight loss, the most common symptom in this group and since it did not increase with disease duration, it is probably the earliest GI symptom of the disease. Fecal incontinence, on the other hand, was infrequent in early stages (with a prevalence similar to that of the general population [46-49]), but the prevalence increased significantly with disease duration.

Not surprisingly, both upper and lower GI symptoms were significant negative predictors of the patients’ nutritional status. The duration of disease also showed a negative association with the nutritional status, which probably reflects the development of multiple GI complications and a marked peripheral polyneuropathy.

All GI symptoms were negatively associated with the EQ-5D Index Score and, expectedly, fecal incontinence had the most profound effect on the patients’ HRQoL. Early disease onset also had a negative impact on the EQ-5D Index Score, which might reflect the higher prevalence of GI symptoms in this group. Not surprisingly, longer duration of disease was associated with a poorer HRQoL as well.

Eighteen percent of the symptomatic patients with TTR mutations had undergone liver transplantation prior to enrollment and these patients have a more stable disease than the non-transplanted. Liver transplantation was included as a variable in the multiple regression analyses and, expectedly, liver transplantation prior to enrollment showed a significant positive association with HRQoL. The transplanted V30M patients were, however, more likely to suffer from GI symptoms, which may be a consequence of post-operative complications.

The mechanisms behind the frequently occurring GI disturbances in patients with hereditary ATTR amyloidosis are not fully elucidated, although malfunction of the autonomic and enteric nervous systems, including a depletion of the GI neuroendocrine cells and interstitial cells of Cajal, seems to be of importance [40,50-55]. Bacterial overgrowth in the small intestine and bile acid malabsorption also contribute to the GI symptoms [56,57].

In conclusion, the present study supports previous data of a high frequency of GI complications, even at early stages, in patients with hereditary ATTR amyloidosis. However, the prevalence of GI symptoms for SSA and mutations predominantly associated with cardiac complications was not different from that reported by the general population. GI symptoms were found to be more common in early-onset patients and increased with disease duration. Both upper and lower GI symptoms were negatively associated with the patients' nutritional status and HRQoL. These findings underscore the importance of a thorough evaluation of the GI function in patients with TTR mutations and should encourage further studies on the phenotypic differences related to genotype, age at onset, and geographic origin.

### Limitations

THAOS is a large international registry involving several sites and physicians with different specialties. The GI symptoms and their duration were registered in THAOS by investigators at the individual sites as reported by the patients. Hence, the registration procedure was not identical across sites and the accuracy of symptom duration data is dependent on the patients’ ability to recollect the onset of their symptoms.

Only 677 out of the 1219 symptomatic patients enrolled had biopsy-proven hereditary ATTR amyloidosis. The patients without biopsy-proven disease were enrolled at sites in endemic areas with a high incidence of the disease, where a histopathological diagnosis is not routinely sought for.

The nutritional status assessed by mBMI is dependent not only on the height-weight ratio, but also on serum albumin. Thus, edema caused by hypoalbuminemia will not lead to an increase in mBMI as it would do in BMI. However, hypoalbuminemia due to liver failure or urinary protein losses will result in a decrease in mBMI and the patients’ liver and kidney function was not accounted for in this analysis.

GI symptoms are common side effects of several drugs and, unfortunately, we had no possibility to adjust for the patients’ medication in this study.

## Abbreviations

ATTR: Transthyretin amyloid; CI: Confidence interval; I68L: Isoleucine substituted for leucine at position 68; FAC: Familial amyloid cardiomyopathy; FAP: Familial amyloid polyneuropathy; GI: Gastrointestinal; HRQoL: Health-related quality of life; L111M: Leucine substituted for methionine at position 111; LTx: Liver transplant; mBMI: Modified body mass index; NSAID: Non-steroidal anti-inflammatory drug; OR: Odds ratio; SCA: Senile cardiac amyloidosis; SSA: Senile systemic amyloidosis; THAOS: The Transthyretin Amyloidosis Outcomes Survey; TTR: Transthyretin; V30M: Valine substituted for methionine at position 30; V122I: Valine substituted for isoleucine at position 122; Wt: Wild-type.

## Competing interest

Rajiv Mundayat and Onur N. Karayal are employees of and hold stock options in Pfizer Inc. Ole B. Suhr is chairman of the THAOS registry, which is sponsored by Pfizer Inc, and is currently participating in clinical trials sponsored by Pfizer Inc and Alnylam Pharmaceuticals. He has also been a member of expert committees for Pfizer Inc and Alnylam Pharmaceuticals and has served as lecturer at meetings and educational activities sponsored by Pfizer Inc. The remaining authors have no competing interests to declare.

## Authors’ contributions

JW contributed to the design of the study and wrote the manuscript. RM contributed to the design of the study, performed the statistical analyses and revised the manuscript. OK contributed to the design of the study and revised the manuscript. IA and PK participated in the planning of the study, in data analysis and in the revision of the manuscript. OBS conceived of the study and participated in its design and coordination. All authors read and approved the final manuscript.
